# Prevalence of *Staphylococcus aureus* Infections in the Implantation of Orthopedic Devices in a Third-Level Hospital: An Observational Cohort Study

**DOI:** 10.3390/pathogens13080620

**Published:** 2024-07-26

**Authors:** Roberto Renan Albavera-Gutierrez, Manuel A. Espinosa-Ramos, Ernesto Rebolledo-Bello, Francisco Javier Paredes-Herrera, Daniel Carballo-Lucero, Omar Esteban Valencia-Ledezma, Carlos Alberto Castro-Fuentes

**Affiliations:** 1 Traumatology and Orthopedics Unit, Hospital Regional de Alta Especialidad de Ixtapaluca, IMSS-BIENESTAR. Calle Gustavo E. Campa 54, Col. Guadalupe Inn, Alcaldía Álvaro Obregón, Ciudad de México C.P. 01020, Mexico; rrag82@hotmail.com (R.R.A.-G.); espinosaramosmanuelalejandro@gmail.com (M.A.E.-R.); rempo8911@gmail.com (E.R.-B.); pa263432@uaeh.edu.mx (F.J.P.-H.); dcarballo@uabc.edu.mx (D.C.-L.); 2 Research Unit, Hospital Regional de Alta Especialidad de Ixtapaluca, IMSS-BIENESTAR. Calle Gustavo E. Campa 54, Col. Guadalupe Inn, Alcaldía Álvaro Obregón, Ciudad de México C.P. 01020, Mexico; esteban84valencia@gmail.com

**Keywords:** *Staphylococcus aureus*, orthopedic implant, infection, postoperative

## Abstract

Using orthopedic devices or prosthetic joints to treat various conditions is expected in a Traumatology and Orthopedics Unit. Recently, the materials used to build these different devices have evolved; however, pathogens can still infect these materials. Additionally, the immune system has limitations when defending against these pathogens, which results in bacterial infections like *Staphylococcus aureus*, *Methicillin-susceptible Staphylococcus aureus* (*MSSA*) and *Methicillin-resistant Staphylococcus aureus* (*MRSA*). A total of 276 patients who attended the Traumatology and Orthopedics Unit of our hospital from 1 June 2018 to 1 June 2019, were included in the present study. Our study analyzed the incidence of *S. aureus* and other bacterial pathogens in the surgical sites of patients with orthopedic implants, as well as the most used types of implants and implant materials. The specimens obtained from the surgical sites of the patients were cultured in anaerobic and aerobic media for subsequent identification using their phenotypic characteristics. Subsequently, antibiotic susceptibility tests were performed to establish the appropriate treatment. The primary pathogens identified were *Staphylococcus aureus* (26.4%), followed by *Escherichia coli* (21.0%) and *Staphylococcus epidermidis* (15.8%). The most commonly used implants were plates (41.7%), followed by endomedullary nails (20%), Kirschner wires (14.1%), and fixators (10.1%). As for the anatomical regions of the implants, the most frequent sites were the legs, followed by the thighs, wrists, and ankles. The pathogens were more susceptible to ciprofloxacin (95%), clindamycin (89%), and cefotaxime (86%). *S. aureus* is the primary infectious agent in our hospital, with an incidence of 26.4% after the placement of orthopedic implants. Although its incidence was lower compared to other tertiary hospitals, it is necessary to improve aseptic techniques in such a way as to reduce the incidence of this pathogen further.

## 1. Introduction

Orthopedic surgeries often involve implanting a foreign body, such as a prosthetic joint, joint components, or others, to stabilize bone structures or repair fractures. These implants can cause infection by direct contamination or by the hematogenous dissemination of microorganisms that are part of the human microbiome and that are found in the intestine, oral cavity, and skin [1].

Infections associated with orthopedic devices usually last for two years, from diagnosis to resolution, and sometimes their treatment is unsuccessful. Orthopedic implants include prosthetic joints, fixation devices, nails, screws, wires, pins, and plates [1,2].

The incidence of infections is low in orthopedic surgeries. However, the demand for surgery requiring orthopedic implants has increased in recent years. In these interventions, the implanted foreign bodies are highly susceptible to bacterial infections due to their hosts being compromised by a phenomenon known as frustrated phagocytosis [2]. Therefore, any implanted orthopedic device could cause infection during the period of bacteremia. It is estimated that the risk of infection after internal fixation ranges from 0.4 to 16.1%, depending on the fracture type.

Among the main etiological agents identified in this type of infection, *Staphylococcus aureus* and Methicillin-resistant *Staphylococcus* (*MRSA*) are the most frequent, followed by bacteria belonging to the *Enterobacteriaceae* family, *Propionibacterium acnes*, and *S. epidermidis*, which complicate the resolution of infection [3,4].

According to previous studies, the incidence of *S. aureus* infection after orthopedic implant placement is 1.13%; 0.68% at the surgical site and 0.28% in the bloodstream. Among these cases, the incidence of *MRSA* was 46%. By culture, *S. aureus* was identified, at the surgical site in 48.0% of cases and in the bloodstream in 35.0% [5]. Another study conducted in a French hospital determined that the incidence of *S. aureus* was 0.7%. The authors identified that the risk factors associated with *S. aureus* infections were mainly active smoking and the index of the national surveillance system of nosocomial infections (95% CI) [6]. On the other hand, [7] they identified the weighted cumulative incidence of *S. aureus* at the surgical site and in the bloodstream to be at 2.55% for nasal carriers and 0.52% for non-carriers.

Currently, there is no established protocol for diagnosing acquired infections after orthopedic implant surgery. Thus, their diagnosis tends to be based on clinical examination, laboratory tests, imaging, and cultures. In addition, the incidence of acquired infections in this setting is unknown [3,4].

Therefore, this study aimed to determine the prevalence of *Staphylococcus aureus* postoperative infections associated with implanted material in orthopedic interventions in the Traumatology and Orthopedics Unit of Hospital Regional de Alta Especialidad de Ixtapaluca, State of Mexico, Mexico.

## 2. Materials and Methods

### 2.1. Patients

A total of 276 patients who attended the Traumatology and Orthopedics Unit of our hospital from 1 June 2018 to 1 June 2019, were included in the present study. The selection criteria were as follows:

### 2.2. Inclusion Criteria

- Patients of both sexes (male and female);
- Patients with a wide range of ages;
- Patients undergoing surgery that required the placement of orthopedic implants.

### 2.3. Exclusion Criteria

- Patients undergoing orthopedic surgery who did not require implants or orthopedic devices.

### 2.4. Orthopedic Implants

A patient was considered to meet the selection criteria for the present study if they were treated at our Traumatology and Orthopedic Unit and underwent surgery requiring an orthopedic implant. The placement of plates, endomedullary nails, Kirchner pins, fixators, wires, partial prostheses, screws/rods, total prostheses, and anchors were considered orthopedic implants. The anatomical regions where the implants mentioned above were placed were the legs, thighs, wrists, ankles, forearms, elbows, hips, arms, knees, hands, spine, pelvis, acetabulum, and feet.

### 2.5. Infections in Orthopedic Implants

Specimens of secretions and tissue from the surgical injury site, with prior surgical lavage of the wound or fracture, were plated on blood agar (Becton, Dickinson and Company, Sparks, MD, USA) for the general isolation of infectious agents. Gram-positive and Gram-negative isolates were subsequently identified by their phenotypic characteristics and Gram staining. In cases of Gram-negative isolates, these were plated on McConkey agar (Becton, Dickinson and Company, MD, USA), while Gram-positive isolates were plated on Salmannitol agar (Becton, Dickinson and Company, MD, USA). The cultures were incubated at 37 °C for a period of 48 h. To evaluate the presence of anaerobic microorganisms, they were plated on BD GasPak EZ Anaerobe Container System Sachets (Becton, Dickinson and Company, MD, USA) following the manufacturer’s instructions.

### 2.6. Antimicrobial Susceptibility Testing

Once the pathogens were identified, antimicrobial susceptibility tests were performed. The pathogen susceptibility assessment was performed with VITEK^®^2 Compact (bioMMérieux, Inc., Durham, NC, USA), following the manufacturer’s instructions. Because this automated equipment uses different types of electronic cards for processing, the concentrations of the antibiotics used are variable. However, susceptibility was assessed using ciprofloxacin, ceftriaxone, trimethoprim-sulfamethoxazole, levofloxacin, amikacin, meropenem, vancomycin, cefotaxime, and clindamycin.

### 2.7. Treatment

When the sample taken from the surgical site had a positive culture result for anaerobic or aerobic microorganisms, antibacterial treatment was prescribed. In addition, the results of the antibacterial susceptibility tests were considered; therefore, the treatment time for each patient was different. The antibacterials used were levofloxacin, meropenem ciprofloxacin, clindamycin, cefotaxime, ceftriaxone, trimethoprim-sulfamethoxazole, amikacin, and vancomycin.

### 2.8. Statistical Analysis

A database was built with input from the patients who met the inclusion criteria, including age, gender, comorbidities, nutritional status, anatomical region, implant type, implant material, infection development, isolated pathogen, treatment, and antibiotic therapy. In addition, a descriptive analysis was performed to determine the data’s percentages, means, and standard deviation. The datasets used and analyzed during the current study are available from the corresponding author upon reasonable request.

## 3. Results

### 3.1. Patients

The average age of patients was 37.1 ± 22.2 years old. The age groups most affected by fractures were 18–40 years old (43.1%), followed by 41–60 years old (20.3%), with a higher incidence in males (67.4%). Among the main comorbidities in the patients, obesity and systemic arterial hypertension were the most frequent, at 30.4% and 12%, respectively (Table 1).

### 3.2. Orthopedic Implants

**Figure 1:**
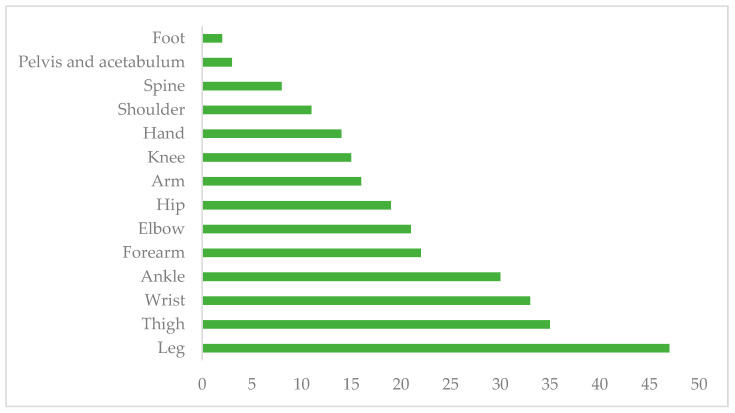
Implants, by anatomical region, performed during the study period.

**Figure 2:**
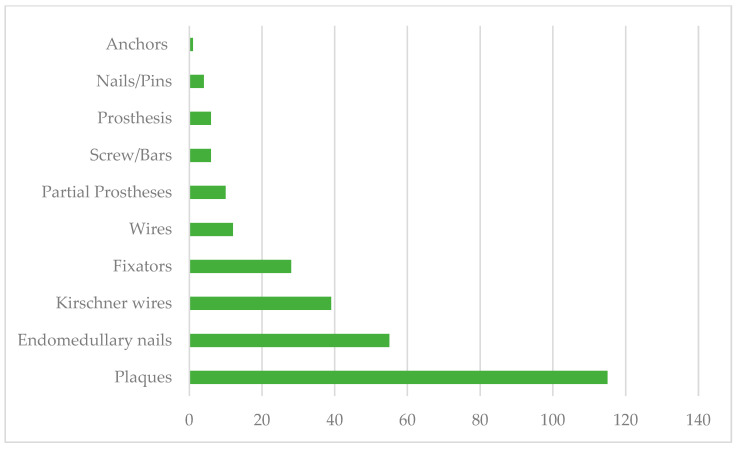
Types of implants used in the Traumatology and Orthopedics Unit.

The orthopedic implants were made of stainless steel (97.2%), titanium (2.1%), and ceramic (0.7%). As for the anatomical regions of the implants, the most frequent were the legs, followed by the thighs, wrists, and ankles (Figure 1). Regarding the type of implant, the most frequently used were plaques (41.7%), endomedullary nails (20%), Kirschner wires (14.1%), fixators (10.1%), wire (3.6%), partial prostheses (3.6%), screws/bars (2.2%), total prostheses (2.2%), screws (1.4), and anchors (0.4%) (Figure 2).

### 3.3. Orthopedic Implant Infections

**Figure 3:**
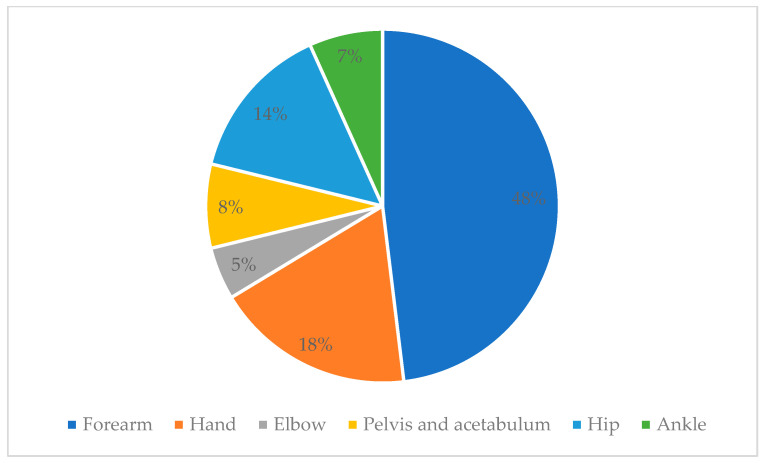
Frequency of infection according to the anatomical region intervened in.

Of the total number of patients, 19 (6.8%) presented with an infection associated with the placement of an orthopedic implant. The male population was more affected than the female population, with a statistical significance of *p* = 0.0035 (Table 2). It is worth mentioning that no significance was found between the frequency of infections and age, comorbidities, or nutritional status. In total, 63.2% of the population had some comorbidity. The main comorbidities identified within the group of infected patients were systemic arterial hypertension (5.2%), diabetes mellitus (15.7%), and overweight and obesity (47.3%). At the same time, the highest frequency of infection occurred in the legs (32%), thighs (21%), and hands (16%) (Figure 3).

**Figure 4:**
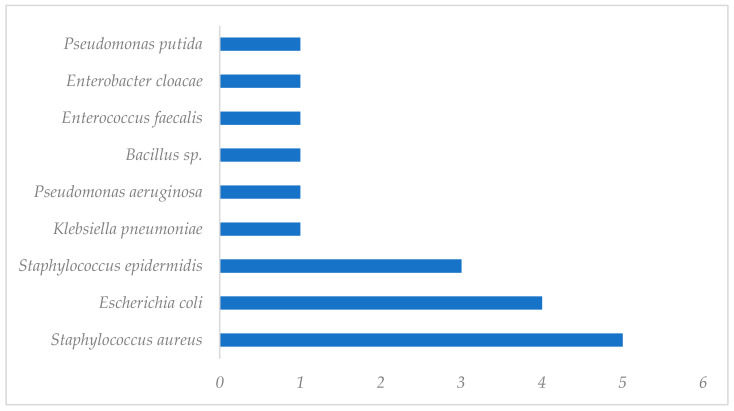
Etiological agents identified in patients who presented with infections after orthopedic implants.

At the Hospital Regional de Alta Especialidad de Ixtapaluca, an infection incidence of 12.9% was identified. The most predominant etiological agents identified in the infections of patients with orthopedic implants were *Staphylococcus aureus* (26.4%), followed by *Escherichia coli* (21.0%), *Staphylococcus epidermidis* (15.8%), *Klebsiella pneumoniae* (5.2%), *Pseudomonas aeruginosa* (5.2%), *Staphylococcus sciuri* (5.2%), *Bacillus* sp. (5.2%), *Enterococcus faecalis* (5.2%), *Enterobacter cloacae* (5.2%), and *Pseudomonas putida* (5.2%) (Figure 4).

### 3.4. Susceptibility to Antimicrobials

The antimicrobial susceptibility testing of the identified pathogens at the wound or fracture site revealed that *S. aureus* was sensitive to ciprofloxacin, cefotaxime, ceftriaxone, and vancomycin primarily and resistant to clindamycin, levofloxacin, and trimethoprim-sulfamethoxazole. *E. coli* was sensitive to ceftriaxone, ciprofloxacin, and amikacin; two patients were resistant to the latter antibiotic. *S. epidermidis* was found to have varying susceptibility to clindamycin, levofloxacin, trimethoprim-sulfamethoxazole, ciprofloxacin, vancomycin, and cefotaxime, with one case of resistance to ciprofloxacin, vancomycin, and cefotaxime. *K. pneumoniae* was only sensitive to clindamycin, ciprofloxacin, levofloxacin, and vancomycin. Likewise, *E. cloracae* showed sensitivity to amikacin, ciprofloxacin, and ceftriaxone. *P. aeruginosa* showed resistance and sensitivity, in one patient, to amikacin and ciprofloxacin, respectively. Regarding the *Enterococcus faecalis* isolate, it was resistant to ceftriaxone and sensitive to amikacin and ciprofloxacin. Finally, *P. putida* showed resistance to levofloxacin and sensitivity to amikacin and ciprofloxacin (Table 3).

### 3.5. Treatment

**Figure 5:**
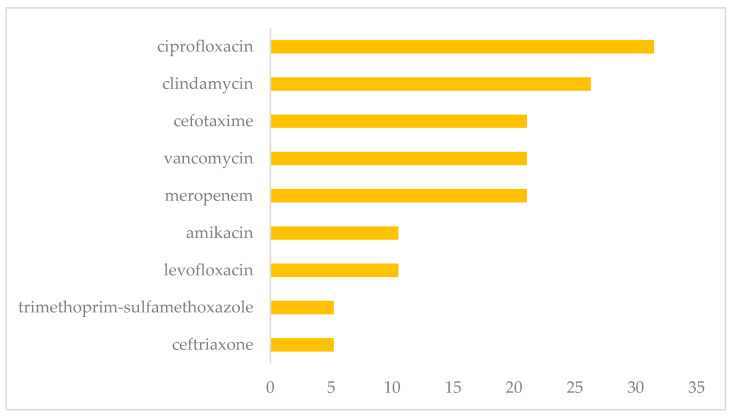
Antimicrobial treatment prescribed to patients with infections after placing orthopedic implants at the HRAEI.

Antibiotic treatment was established based on the susceptibility results. Thus, the main antimicrobials used were ciprofloxacin (31.5%), to treat infections caused by all identified etiologic agents (*S. aureus*, *E. coli*, *S. epidermidis*, *K. pneumonia*, *P. aeruginosa*, *E. faecalis*, *E. chloracae*, *P. putida*), followed by clindamycin (26.3%), for the treatment of infections caused by *S. aereus*, *S. epidermidis*, and *K. pneumoniae*, and cefotaxime (21.05%) for *S. aereus* and *S. epidermidis*. Additionally, the administration of vancomycin was considered; however, cases of resistance in *S. epidermidis* were identified (Figure 5).

## 4. Discussion

The present work identified the prevalence of *Staphylococcus aureus* infections resulting from the placement of orthopedic implants at the Hospital Regional de Alta Especialidad de Ixtapaluca from 1 June 2018 to 1 June 2019.

Men were the group with the highest incidence of postoperative infections, particularly those aged 18–40 years old. This group had comorbidities such as obesity and systemic arterial hypertension. The most frequent anatomical region of implantation was the legs, followed by the thighs, wrists, and ankles. The most commonly used types of implants were plaques, endomedullary nails, Kirschner wires, and fixators, followed by wires, partial prostheses, bars, total prostheses, screws, and anchors. According to different studies, the implanted biomaterials play a crucial role in the success of orthopedic procedures. Pure titanium and its alloys are the preferred materials for permanent implants in contact with bone. However, infections related to implants can hinder a successful outcome. The most critical pathogenic event in developing an infection in biomaterials is biofilm formation, which begins immediately after bacterial adhesion [3,8].

Two hundred forty-six cases were studied, of which nineteen (6.8%) presented with an infection associated with the implantation of orthopedic devices. The main anatomical regions that were infected were the forearm, hand, elbow, pelvis, acetabulum, hip, and ankle. Infectious agents were identified by means of the macromorphological and micromorphological characteristics. The main infectious agents in the orthopedic implants of these HRAEI patients were *Staphylococcus aureus* (26.4%)*, Escherichia coli* (21.0%), and *Staphylococcus epidermidis* (15.8%). In contrast, reports from other high-specialty units showed infections primarily caused by *Escherichia coli* (59%), followed by *Staphylococcus aureus* (22%) [3]. It is important to mention that in studies where *S. aureus* was identified as the main etiological agent of surgical site infections, *MRSA* represented 46% of the *S. aureus* isolates [5,6,7].

It is known that bacterial colonization, biofilm formation around implants, and invasion into dense skeletal tissue matrices are difficult to treat and can lead to implant failure and osteomyelitis. These complications require major revision surgeries and prolonged antibiotic therapies, which are associated with high treatment costs, morbidity, and even mortality. Therefore, it is essential to implement adequate aseptic techniques in surgical interventions and take preventive measures to reduce patient complications. If these steps are taken, treatment costs would be considerably reduced [1,4,9,10].

Currently, molecular biology techniques allow for the identification of these organisms to implement appropriate treatments. The treatment for patients with identified infections includes antibiotic therapy and surgical debridement. Treatment for postoperative infections is established empirically by administering penicillin (oxacillin, cloxacillin, or dicloxacillin) or a first-generation cephalosporin such as cefazolin. However, up to 20% of *Staphylococcus aureus* strains are resistant to methicillin, so the antibiotic of choice is vancomycin [1,11]. However, according to a recent clinical trial in which vancomycin and cefazolin were used as prophylaxis to prevent surgical site infections among patients with knee, hip, and shoulder arthroplasty, the addition of vancomycin was not superior to a placebo administered in an aleatory manner. Furthermore, adverse effects such as hypersensitivity reactions and acute kidney injury can occur [12]. However, the application of vancomycin powder has been shown to decrease the proportion of *S. aureus* in cultures taken from infected surgical sites where fractures were treated surgically [13].

In our hospital unit, the main antibiotics used were ciprofloxacin, clindamycin, cefotaxime, and vancomycin. The other treatment of choice for *Staphylococcus aureus* infections is rifampicin because it has demonstrated great activity on adherent *Staphylococcus* when combined with beta-lactams, glycopeptides, fluoroquinolones, minocycline, cotrimoxazole, or fusidic acid [14].

As part of the treatment for these types of patients, whether to keep or remove the implant must be determined. For implant removals, combinations with rifampicin for *Staphylococcus* spp. infections are recommended [14]. However, it is advantageous to evaluate the resistance of these agents before starting treatment, since some isolates may not be susceptible to quinolones combined with rifampin [15]. An essential consideration for orthopedic surgeons is the presence of Methicillin-resistant *Staphylococcus* (*MRSA*); therefore, screening and eliminating these organisms upon hospital admission is recommended. However, this is only done when there are risk factors, as per current American guidelines [16]. Nonetheless, evidence suggests that postoperative infections in prosthetic implants can be reduced by detecting and eliminating microorganisms in the prostheses and patients. The prosthesis can be saved after infection if clinical manifestations are present for ≤10 days, since the microorganisms are susceptible to oral antibiotics [17]. The perioperative use of mupirocin to prevent *MRSA* infections in orthopedic surgical sites during the insertion of metallic prostheses or fixation has also been evaluated. A group of 2178 patients were treated with nasal mupirocin for five days, in addition to a 2% triclosan bath before surgery. A statistically significant decrease in the incidence of *MRSA* was reported. Meanwhile, the point prevalence of the nasal carriage of *MRSA* decreased after treatment from 38% to 23%. It is worth mentioning that no *S. aureus* isolates with high resistance to mupirocin were found.

*S. aureus* is a colonizer of the nose and skin, so it represents a risk factor for infection in a patient undergoing surgery for the placement of an orthopedic device. Thus, adequate aseptic techniques, as well as identifying and eliminating nasal *S. aureus* by applying mupirocin in combination with a chlorhexidine bath prior to surgery, contribute to reducing infections by this microbial agent, including strains that are susceptible and resistant to methicillin [18,19,20].

It is worth mentioning that, despite the high frequency of *Staphylococcus aureus* infections in orthopedic surgical sites, other rare *Staphylococcus* agents, such as *S. lugdunensis*, have been reported. This etiological agent infects fracture fixation devices. The clinical manifestation of *S. lugdunensis* infections includes purulent discharge, pain, local inflammation, and fever. In a study conducted by Seng et al. [21], *S. lugdunensis* was identified as the etiological agent in this type of infection in the tibia. The treatment of choice was a combination of surgery and antimicrobial therapy, including surgical debridement, antibiotics, and osteosynthesis.

## 5. Conclusions

*Staphylococcus aureus* is the primary etiological agent of infections in orthopedic implants in our hospital (26.4%). However, a low incidence of postoperative infections was identified among the patients undergoing surgery in the Traumatology and Orthopedics Unit. Although the incidence was lower in our institution compared to other tertiary hospitals, it is necessary to improve aseptic techniques to further reduce the incidence of this pathogen.

The male population was the one that presented with a higher incidence of postoperative infections. Postoperative infections were associated with the most commonly used implants, such as plates, endomedullary nails, and Kirschner wire, and the most common intervened-upon anatomical sites were the legs, thighs, and hands.

Antimicrobial susceptibility testing of the identified pathogens from the wound and fracture sites revealed that *S. aureus* was primarily sensitive to ciprofloxacin, cefotaxime, ceftriaxone, and vancomycin and resistant to clindamycin, levofloxacin, and trimethoprim-sulfamethoxazole. Thus, the antibiotic treatment of choice for infections caused by this pathogen was ciprofloxacin.

## Figures and Tables

**Table 1:** Somatometry of the patients included in the study.

Variable	Frequency (n)	Percentage
Sex		
Female	186	67.4%
Male	90	32.6%
Age group		
<18 years old	53	19.25%
18–40 years old	119	43.1%
41–60 years old	56	20.3%
>61 years old	48	17.4%
Total	276	100%
Comorbidities (Chronic degenerative diseases)		
SAH	33	12.0%
DM 2	5	1.8%
SAH + DM 2	8	2.9%
SAH + DM 2 + RA	1	0.4%
HIV	1	0.4%
Total	48	17.5%
Nutritional status (kg/m^2^)		
BMI (<18.5)	1	0.4%
BMI (18.5–24.9)	120	48.0%
BMI (>25)	53	21.2%
Total	246	100%

SAH: systemic arterial hypertension; DM 2: Diabetes Mellitus Type 2; RA: Rheumatoid Arthritis; HIV: Human Immunodeficiency Virus; BMI: body mass index.

**Table 2:** Percentage distribution of infections associated with implant placement according to sex, age, comorbidities, and nutritional status.

Variable	Postsurgical Infections		Value
	Yes = 6.88%	No = 93.11%	
Sex			
Female	5 (26.3%)	14 (73.6%)	8.53
Male	14 (73.6%)	5 (26.3)	0.0035
Age groups			
Less than 40 years old	8 (42.1%)	11 (57.8%)	0.95
41 and older	11 (57.8%)	8 (42.1%)	0.3303
Comorbidities (Chronic degenerative diseases)			
Yes	9 (47.3%)	10 (52.6%)	0.11
No	10 (52.6%)	9 (47.3%)	0.7456
Nutritional status			
BMI (18.5–24.9)	9 (47.3%)	10 (52.6%)	0.11
BMI (<18.5 and >25)	10 (52.6%)	9 (47.3%)	0.7456

**Table 3:** Patients with resistance and sensitivity to infectious agents identified during orthopedic implant placement.

Pathogen	Antibiotic	Patients with Resistance n (%)	Patients with Sensitivity n (%)
*Staphylococcus aureus* n = 5	clindamycin	1 (20%)	4 (80%)
	ciprofloxacin	0	5 (100%)
	levofloxacin	1 (20%)	4 (80%)
	trimethoprim-sulfamethoxazole	1 (20%)	4 (80%)
	cefotaxime	0	5 (100%)
	ceftriaxone	0	5 (100%)
	vancomycin	0	5 (100%)
*Escherichia coli* n = 4	amikacin	2 (50%)	2 (50%)
	ciprofloxacin	0	4 (100%)
	ceftriaxone	0	4 (100%)
*Staphylococcus epidermidis* n = 3	clindamycin	0	3 (100%)
	ciprofloxacin	1 (33.3%)	2 (66.6%)
	levofloxacin	0	3 (100%)
	vancomycin	1 (33.3%)	2 (66.6%)
	cefotaxime	1 (33.3%)	2 (66.6%)
	trimethoprim-sulfamethoxazole	0	3 (100%)
*Klebsiella pneumoniae* n = 1	clindamycin	0	1 (100%)
	ciprofloxacin	0	1 (100%)
	levofloxacin	0	1
	vancomycin	0	1
*Pseudomonas aeruginosa* n = 1	amikacin	1 (100%)	0
	ciprofloxacin	0	1 (100%)
*Enterococcus faecalis* n = 1	amikacin	0	1 (100%)
	ciprofloxacin	0	1 (100%)
	ceftriaxone	1 (100%)	0
*Enterobacter cloracae* n = 1	amikacin	0	1 (100%)
	ciprofloxacin	0	1 (100%)
	ceftriaxone	0	1 (100%)
*Pseudomonas putida* n = 1	amikacin	0	1 (100%)
	ciprofloxacin	0	1 (100%)
	levofloxacin	1 (100%)	0

## Data Availability

Data are contained within the article.
